# Understanding chemotherapy treatment pathways of advanced colorectal cancer patients to inform an economic evaluation in the United Kingdom

**DOI:** 10.1038/sj.bjc.6605766

**Published:** 2010-07-27

**Authors:** F H Shabaruddin, R A Elliott, J W Valle, W G Newman, K Payne

**Affiliations:** 1 Health Sciences – Economics, School of Community Based Medicine, The University of Manchester, 1st Floor, Jean McFarlane Building, Oxford Road, Manchester M13 9PL, UK; 2 School of Pharmacy, The University of Nottingham, University Park, Pharmacy School Building, Nottingham NG7 2RD, UK; 3 Department of Medical Oncology, Christie NHS Foundation Trust, Manchester M20 4BX, UK; 4 Genetic Medicine, St Mary's Hospital, Manchester Academic Health Sciences Centre, The University of Manchester, Manchester M13 0JH, UK

**Keywords:** colorectal cancer, treatment pathways, economic evaluation

## Abstract

**Background:**

Accurate description of current practice within advanced colorectal cancer (CRC) specialties were needed to inform an economic evaluation of the UGT1A1 pharmacogenetic test for irinotecan in the United Kingdom.

**Methods:**

The study was based on a literature review and elicitation of expert opinion. The expert panel comprised 44 consultant oncologists in NHS Hospital Trusts across England.

**Results:**

Ten first-line, 10 second-line and 12 third-line chemotherapy regimens were reported, reflecting wide variations in treatment pathways. Predominant pathways emerged with: first-line treatment with oxaliplatin-based regimens, second-line treatment with irinotecan-based regimens and third-line treatment with mitomycin-based regimens. Experts estimated the frequency of febrile neutropaenia 8.4% (95% CI: 6.7–10.0), septic neutropaenia 4.7% (95% CI: 3.4–6.0) and severe diarrhoea 13.1% (95% CI: 10.8–15.5). Approaches for the clinical management of neutropaenia within the NHS were described.

**Conclusions:**

This study identified wide variations in the clinical management of advanced CRC patients. Descriptions of current treatment pathways are necessary for economic evaluations. Variations in clinical practice must be reflected in the model to ensure the findings from an economic evaluation of UGT1A1 testing are sufficient to inform policy regarding the cost-effective use of NHS resources.

Irinotecan is a chemotherapeutic agent used in the treatment of colorectal cancer (CRC) and lung cancer. In the United Kingdom, irinotecan is licensed for first- and second-line chemotherapy for advanced CRC patients (electronic Medicines Compendium, 2009). The main dose-limiting toxicities from irinotecan chemotherapy are severe neutropaenia and diarrhoea.

Irinotecan is a pro-drug that is activated into SN-38 in the liver, and then converted by hepatic and extrahepatic UDP-glucuronosyltransferase (UGT) enzymes into an inactive compound, SN-38G (Rivory and Robert, 1995). UGT1A1 enzyme is the major isozyme involved in this conversion (Nagar and Blanchard, 2006). In Caucasian populations, UGT1A1^*^1 and UGT1A1^*^28 genetic variants accounts for 98–99% of UGT1A1 polymorphisms (Palomaki *et al*, 2009). Individuals homozygous for UGT1A1^*^28 have been found to have increased risk of irinotecan-induced severe neutropaenia (Ando *et al*, 2000; Innocenti *et al*, 2004; Rouits *et al*, 2004) and severe diarrhoea (Ando *et al*, 2000; Marcuello *et al*, 2004; Ferraldeschi *et al*, 2009).

UGT1A1 pharmacogenetic test is a genotyping assay that can predict the risk of irinotecan-related neutropaenia and diarrhoea by providing information on patients' genotype, which can be used to stratify patients into low-, intermediate- or high-risk groups, defined in terms of the risk of severe neutropaenia or diarrhoea (Palomaki *et al*, 2009). Some evidence suggests that the association between UGT1A1^*^28 and haematological toxicity depends on the irinotecan dose, with a higher risk of toxicity for UGT1A1^*^28 homozygous individuals on medium- or high-dose irinotecan regimens (Hoskins *et al*, 2007). In the United States, the Food and Drug Administration advised that irinotecan dosing should be reduced for individuals homozygous for UGT1A1^*^28 allele (United States Food and Drug Administration, 2005) but do not specify the degree of dose reduction. A prospective study (Toffoli *et al*, 2006) found that CRC patients homozygous for the UGT1A1^*^28 alleles may have increased clinical benefit and tumour response to irinotecan-based chemotherapy. This increased benefit was only statistically significant for surrogate outcomes, which were progressive disease and stable disease, and did not result in significant overall survival advantage. A pharmacogenetic test that provides information on UGT1A1^*^28 genetic variation may have a role in individualising irinotecan dosing to reduce the incidence of severe neutropaenia and diarrhoea, thereby improving the safety profile of irinotecan-based regimens. Currently there are no data from randomised controlled trials to support the use of UGT1A1 testing to guide irinotecan dosing.

In the United States, the Invader assay was marketed to test for UGT1A1 status, costing between USD$250 and $500 a test (Anonymous, 2006). Since January 2009, UGT1A1 testing has been offered by a UK NHS laboratory at a fee of £30 per test. The acquisition cost of the test is only one of many factors driving the costs associated with its use. Altered clinical pathways arising from the use of the pharmacogenetic test may lead to additional NHS resource use downstream, and therefore needs to be assessed within the broader framework of health economic evaluation. Some evidence suggests that managing severe neutropaenia and diarrhoea is expensive, time consuming and labour intensive (Hassett *et al*, 2006; Maroun *et al*, 2007). Using the UGT1A1 test results to inform irinotecan dosing by stratifying patients into different risk groups will impact upon clinical treatment decisions, patient outcomes and NHS resources. In 2009, a House of Lords report on genomic medicine highlighted that many genetic tests are diffusing into practice without proper assessments of their clinical utility or validity (House of Lords Science and Technology Committee, 2009).

The economic evaluation framework enables assessment of an intervention's incremental clinical benefits and costs over existing treatments. Sculpher *et al* (1997) recommended an iterative approach to economic evaluation, with an assessment in the early life of the intervention, and supplemented by further evaluations as and when new evidence become available. Because the UGT1A1 test is a new potential addition to practice and the level of evidence required to achieve regulatory approval for this type of technology is low (Payne, 2009), current empirical evidence on its costs and benefits in clinical practice is likely to be limited. To date, two economic evaluations of using UGT1A1 testing to inform irinotecan prescribing have been published (Obradovic *et al*, 2008; Gold *et al*, 2009). However, both studies were based on the US health-care system and assumed irinotecan regimens and care pathways that are not relevant to current UK practice.

The results of a UK relevant economic evaluation will aid decision-makers commissioning health-care services to make an informed decision on whether the UGT1A1 pharmacogenetic test is a clinically and cost-effective use of scarce NHS resources. Systematic evaluation of all relevant evidence within an economic model will also allow explicit consideration of the expected value of additional information generated by future research, for example a prospective trial evaluating the clinical utility of UGT1A1 test, and whether that would outweigh the cost of undertaking the trial in the first place (Sculpher and Claxton, 2005).

To construct the pathways for an economic model, it is necessary to describe current clinical practice in the setting that the UGT1A1 test will be used. This includes a description of commonly used chemotherapy regimens, clinical management of patients and incidence and management of adverse drug events (ADEs). In general, there is a paucity of data describing current clinical practice in UK cancer specialties, making evaluation of new interventions challenging. The only previous study that described clinical practice of advanced CRC specialties in the United Kingdom (Seymour *et al*, 1997) found substantial diversity of practice among experts in the field. At the time the study was conducted, chemotherapy options within advanced CRC specialties were limited to 5-fluorouracil (5-FU)-based regimens.

An extensive selection of chemotherapy treatment options available within current practice, accompanied by a rapidly changing evidence base, result in wide variations in prescribing practice and difficulties in describing current clinical practice in terms of the main treatment pathways within oncology specialties. The structure of the economic model must accurately reflect the anticipated place of the new intervention in clinical practice.

This study aimed to inform an economic evaluation of UGT1A1 pharmacogenetic test by identifying the predominant advanced CRC treatment pathways in the UK NHS, focusing specifically on the composition and placement of commonly used irinotecan-based regimens, the clinical management of patients on irinotecan-based chemotherapy, the frequency of irinotecan-related diarrhoea and neutropaenia, and the resource implications associated with the management of patients experiencing irinotecan-related neutropaenia. It also aimed to explore early perceptions about the value of UGT1A1 pharmacogenetic testing in CRC specialties by eliciting consultants' current use and future preferences for the test. The results from this study will inform an economic evaluation of the UGT1A1 pharmacogenetic test, which will ensure that the model design and subsequent results are relevant to decision-makers in the NHS and are sufficient to inform policy regarding the cost-effective use of NHS resources.

## Methods

Initially, systematic reviews of the literature were planned, using electronic search strategies and methods described in The Cochrane Handbook of Systematic Reviews (The Cochrane Collaboration–Higgins and Green, 2009) to identify publications that report the frequency of irinotecan-related neutropaenia and diarrhoea, describe CRC chemotherapy treatment pathways and describe clinical management of irinotecan-related neutropaenia in the United Kingdom. Attempts at a comprehensive systematic review based on terms relating to toxicity were hampered by insurmountable difficulties in identifying relevant studies. Two main problems were identified. First, key studies of irinotecan-based regimens consist primarily of Phase III clinical trials that cannot be identified by search terms that describe harm from medicines such as ‘adverse reactions’ and ‘neutropaenia or diarrhoea’. Second, attempts to identify studies other than Phase III trials that report the frequency of irinotecan adverse events yielded too many irrelevant publications but also missed key studies because (i) of the many terms to describe harm from medicines and (ii) these studies may not have these terms indexed as keywords. It is well recognised that searches for adverse events are problematic because data are often sparse with various challenges in identifying relevant studies (Craig *et al*, 2009; The Cochrane Collaboration–Higgins and Green, 2009).

The initial review also revealed a paucity of data describing current NHS treatment pathways of CRC chemotherapy and management of neutropaenia. No publications describing NHS patients' current clinical pathways were identified, because trial protocol often dictates clinical practice. The limitations of using this method meant that alternative methods were required to identify the frequency of irinotecan's adverse events and NHS treatment pathways for the economic model.

Two methods were used in parallel: (1) a review of relevant literature identified from a published systematic review and (2) a national survey of expert opinion on current practice within NHS CRC specialties. The study involved three steps. Step 1 identified the range of potential chemotherapy regimens evaluated in published randomised controlled trial (RCTs) for advanced CRC (identified from the published systematic review). Step 2 used the survey to identify current UK practice. Step 3 combined the findings from the literature review and survey to select the published trials relevant to UK practice.

### Review of published RCTs

A published systematic review of chemotherapy treatment regimens for advanced CRC (Golfinopoulos *et al*, 2007) was used to identify publications reporting RCTs that used irinotecan-based chemotherapy. The bibliography of this review was also searched to identify additional studies.

### National survey of expert opinion

Elicitation of expert opinion was required to inform the economic evaluation as other data sources were not available (Philips *et al*, 2006). As there are no national databases available to identify oncology consultants with advanced CRC practice, a postal survey method was developed to identify relevant clinical experts in CRC specialties. The inclusion criteria for the survey were specific: an eligible participant (an ‘expert’) was defined as a consultant oncologist (specialist) who prescribes irinotecan to advanced CRC patients in NHS Hospital Trusts in England. Publicly available websites (Dr Foster and NHS Hospital Trusts) were used to develop a sampling frame of potential clinical experts (*n*=124). This approach has previously been used to successfully identify survey participants (Fargher *et al*, 2007; Wordsworth *et al*, 2008).

The survey questions were informed by discussions with clinical and economic experts and literature review. The survey mainly comprised closed-ended questions, supplemented with free-text response options and was divided into two main sections: (1) descriptions of advanced CRC treatment pathways and irinotecan prescribing practice; and (2) current use and future preferences for pharmacogenetic testing in CRC specialties (survey available on request from corresponding author). The survey results will be used to identify which of the published studies of irinotecan-based regimens are relevant to UK practice.

After University of Manchester Ethics Committee approval and a pilot study of eight consultant oncologists, the Pharmacogenetic testing in Colorectal Cancer specialties survey was posted to experts between May and August 2008. Clinicians were asked to focus on NHS, rather than private, clinical practice when answering the questions to reflect the same perspective as the economic evaluation. After 1 month, a postal reminder was sent to non-respondents and then a further 1 month later a random sample of 33% of non-respondents was telephoned to identify their reason(s) for not participating.

### Analysis

Data on the frequencies of irinotecan-related neutropaenia and diarrhoea that were identified in published RCTs found from the hand search of the literature (the published systematic review and the references) were tabulated. Survey data were summarised with descriptive statistics using SPSS 15.0 (SPSS Inc., Chicago, IL, USA). Clinicians' estimates were weighted against the number of advanced CRC patients seen by them in 2007 as a proxy measure to account for diverse levels of experience and expertise in CRC. To be included in the weighted analysis, respondents must have provided estimates for both the parameter of interest and number of patients. For scale data where respondents indicated less than a certain value (for example, <10%), the mean between zero and that value was inputted for analysis purposes (for example, 5%). If respondents provided data as a range value, then the mean of the range was used. Estimates from the survey on the frequency of ADEs in NHS practice were compared to the incidence data from the identified trials.

## Results

Results are presented in two sections: (1) the findings from the literature review and (2) the results of the national survey. It was necessary to analyse the national survey to inform the final selection of the RCTs relevant to UK practice.

### Literature review of published RCTs

The literature search identified 22 RCTs investigating the use of irinotecan in combination with 5-FU, all of which can be found in the published systematic review (Golfinopoulos *et al*, 2007). Twelve trials used irinotecan and bolus 5-FU regimens, whereas 10 used irinotecan and infusional 5-FU. There are a number of different regimens for irinotecan with infusional 5-FU, including AIO, irinotecan modified de Gramont (IrMdG) and variations of the FOLFIRI regimen.

It was, therefore, necessary to use the findings from the national survey to define the regimen that is relevant to UK practice, which was irinotecan with infusional 5-FU according to the IrMdG regimen as second-line therapy. Of the 10 RCTs that used irinotecan and infusional 5-FU, only 1 (Seymour *et al*, 2007) used IrMdG regimen. There were two other trials that used chemotherapy regimens similar to the UK IrMdG regimen, which was FOLFIRI with high-dose infusional 5-FU (Tournigand *et al*, 2004; Van Cutsem *et al*, 2009). The frequencies of irinotecan-related neutropaenia and diarrhoea from the three trials are summarised in Table 1. In addition, one non-randomised Phase II study (Leonard *et al*, 2002) of IrMdG, which was a pilot study for the FOCUS trial, was found.

All three RCTs reported the frequencies of irinotecan-related grade 3 and 4 neutropaenia and diarrhoea as defined by standard criteria (National Cancer Institute, 2006). Only two (Tournigand *et al*, 2004; Van Cutsem *et al*, 2009) reported the frequency of febrile neutropaenia. None reported the frequency of septic neutropaenia. Febrile and septic neutropaenia differ in severity and clinical management, thus consume different levels of resources, which make their frequencies key parameters for the economic model.

### Elicitation of expert opinion by national survey

**Figure 1:**
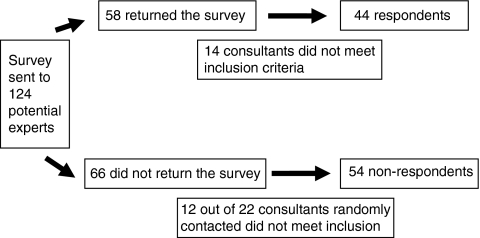
Flow chart describing the sampling frame for the survey.

The final expert panel comprised 44 consultants: 28 clinical oncologists and 16 medical oncologists. Figure 1 illustrates how the eligibility of participants was determined and the final expert panel numbers.

A total of 58 clinicians returned the survey but 14 of these were excluded, as they did not manage CRC patients on irinotecan chemotherapy. One-third of the 66 clinicians who did not return the survey (*n*=22) were randomly selected and contacted by telephone. Of the 22 clinicians contacted, 12 did not treat patients with CRC, which reflects inaccuracies on the public websites. This left an active sample of 98 potential experts and gave a 45% (*n*=44) survey completion rate. There was at least one consultant from each English NHS region and Strategic Health Authority. The mean time since graduation from medical school was 24 years (range: 7–41 years). The mean number of patients seen by each clinician in 2007 was 135 (range: 15–600).

#### Indications for irinotecan-based regimens

All 44 experts use irinotecan-based chemotherapy for palliative treatment of advanced CRC. Only 12 (27%) reported using irinotecan-based regimens as neoadjuvant chemotherapy for potentially resectable cancer. The following data are based on irinotecan regimens used as palliative treatment of advanced CRC.

#### Chemotherapy regimens for advanced CRC patients

To identify the main chemotherapy treatment pathways, we asked clinicians to assign an estimated percentage reflecting the frequency of use for first-, second- and third-line chemotherapy regimens. Ten first-line, 10 second-line and 12 third-line chemotherapy regimens were reported. Oxaliplatin (130 mg m^−2^) in combination with capecitabine (1000 mg m^−2^) was reported to be the most commonly used first-line regimen, followed by OxMdG (oxaliplatin (85 mg m^−2^) with infusional 5-FU according to MdG regimen). Irinotecan regimens were reported to be most frequently used for second-line chemotherapy, with a slight preference for IrMdG (irinotecan (180 mg m^−2^) with infusional 5-FU according to MdG regimen), over irinotecan monotherapy (350 mg m^−2^). For third-line treatment, the survey results suggested a clear preference for using mitomycin (7 mg m^−2^) with either intravenous (protracted infusion 5-FU 300 mg m^−2^) or oral fluoropyrimidine formulation (e.g., capecitabine 1250 mg m^−2^). Table 2 presents the five most commonly used chemotherapy regimens for first-, second- and third-line treatment of advanced CRC.

#### Management of patients on irinotecan regimens

Three areas of managing patients on irinotecan regimens were explored: the clinical setting for administration, indications for stopping treatment and duration of treatment.

- *Clinical setting for administration of irinotecan regimens*: irinotecan regimens were frequently administered in the day case setting (mean 56%, weighted mean 61%), followed by outpatient setting (mean 42%, weighted mean 36%) and inpatient setting (mean 3%, weighted mean 3%). The average time spent in an outpatient clinic was 3.3 h (*n*=25, range 2–8 h, 95% confidence interval (CI) 2.7–3.8 h, mean weighted estimate: 3.5 h).
- *Indications for stopping irinotecan regimens*: there are many potential reasons for stopping chemotherapy, especially in the palliative setting. The majority of respondents (93%) answered these specific questions. The most common reason reported for stopping irinotecan-based chemotherapy was lack of therapeutic response (41%), followed by completion of the specified duration of chemotherapy (25%) and tumour progression despite chemotherapy (21%). When asked for the least likely reasons for cessation of irinotecan-based regimens, 32% of clinicians reported severe chemotherapy-related toxicity, followed by deterioration in patient's clinical condition (27%).
- *Time duration for irinotecan treatment*: on average, clinicians estimated that irinotecan treatment was given for 5.0 months (*n*=13, range 3–6 months, 95% CI 4.2–5.8 months, mean weighted estimate: 5.3 months).

#### Frequency of ADEs due to irinotecan regimens

The experts estimated the percentages of their advanced CRC patients on irinotecan-based regimens who develop these ADEs:

1. Uncomplicated neutropaenia (neutropaenia of any grade not complicated by fever or infection)
2. febrile neutropaenia (neutropaenia complicated by fever ⩾38.5°C without clinically or microbiologically documented infection)
3. septic neutropaenia (neutropaenia with a clinically or microbiologically documented infection)
4. severe diarrhoea (grade 3 or 4 diarrhoea)

**Figure 2:**
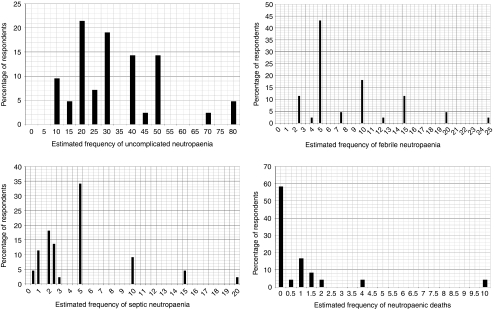
Distributions of unweighted estimated frequencies for uncomplicated, febrile and septic neutropaenia and neutropaenic deaths.

Table 3 presents the experts' estimates on the frequency of ADEs. Figure 2 illustrates the wide ranges of estimated values and distributions of the unweighted estimated frequencies for neutropaenia when plotted against the percentage of respondents.

#### Management of neutropaenia due to irinotecan regimens

Twenty-five experts (57%) stated that they do not treat uncomplicated neutropaenia, whereas 13 (30%) reported outpatient treatment and 3 (7%) treated in day case settings. One expert (2%) reported treating uncomplicated neutropaenia in the inpatient setting. Two clinicians did not answer this question (5%). For complicated neutropaenia (neutropaenia of any grade complicated by fever or infection), 43 clinicians (98%) manage by inpatient treatment. One clinician (2%) did not answer this question. Mean duration of inpatient stay due to neutropaenic events was 4.8 days (*n*=39, range 2–10 days, 95% CI 4.3–5.3 days, mean weighted estimate: 5.1 days).

#### Use of granulocyte colony-stimulating factor

Twenty-three experts (52%) prescribe granulocyte colony-stimulating factor (G-CSF) in their NHS practice whereas 21 (48%) do not. G-CSF was most frequently used to treat septic neutropaenia (17 consultants, 39%), followed by its use as secondary prophylaxis (11 consultants, 25%) where G-CSF is administered to prevent neutropaenic events in patients with previous neutropaenia. Four (9%) consultants used G-CSF to treat febrile neutropaenia and two (5%) to treat uncomplicated neutropaenia. None prescribe G-CSF for primary prophylaxis, where G-CSF is given to prevent neutropaenia in patients with no previous neutropaenia. Some respondents cited lack of funding as the reason for not using G-CSF in their NHS practice, with several reporting prescribing G-CSF only in their private practice.

Sixteen clinicians prescribe standard formulation multiple-dose G-CSF, four prescribe single-dose long-acting formulation, two prescribe both formulations and one did not report formulation used. Mean duration of G-CSF treatment is 4.4 days (*n*=13, range 3–10 days, 95% CI 3.2–5.5 days, mean weighted estimate: 3.9 days).

#### Clinical consequences of neutropaenia

On average, the experts estimated 1 in every 100 patients on irinotecan-based chemotherapy die because of neutropaenic episodes (*n*=24, range 0–10 deaths, 95% CI: 1 death in every 50 patients to 1 death in every 1430 patients, see Figure 2). This estimate for mean number of neutropaenia-related deaths dropped to 1 death in every 140 patients when estimates are weighted for the number of patients seen in 2007.

#### Current use and future preferences for UGT1A1 pharmacogenetic testing

Of the 44 experts, 27% have previously used at least one pharmacogenetic test (such as *KRAS* and DPD tests), whereas 68% had never used a pharmacogenetic test and 5% were uncertain. None of the experts had used the UGT1A1 pharmacogenetic test. When asked about future preferences, 23 clinicians (52%) indicated that if the test were available, they would use UGT1A1 testing to predict risk of irinotecan-related neutropaenia whereas 26 clinicians (59%) would use the test to predict risk of irinotecan-related diarrhoea.

## Discussion

This study identified chemotherapy treatment pathways and clinical management of NHS advanced CRC patients, frequency of irinotecan-related neutropaenia and diarrhoea, clinical management of irinotecan-related neutropaenia, and current use and future preferences for UGT1A1 pharmacogenetic testing. Wide variations in NHS chemotherapy prescribing practice were identified, which reflect the number of potential combinations with known active agents and suggest considerable uncertainty regarding the ideal prescribing regimens and treatment sequence, corresponding with the small number of clinical trials investigating use of sequential chemotherapy (Tournigand *et al*, 2004; Seymour *et al*, 2007). A study (Ferro *et al*, 2008) in the United States, which used data from a nationwide registry and identified eight commonly prescribed CRC regimens based on the component chemotherapy agents, also found variations in the use of CRC chemotherapy regimens. Ferro *et al* (2008) shows that the irinotecan-based regimens used in the United States are not consistent with the UK-relevant regimens identified in this study.

Despite the lack of standardised patient pathways described in the literature, this study identified predominant treatment pathways within NHS CRC specialties with: first-line treatment with oxaliplatin-based regimens, second-line treatment with irinotecan-based regimens and third-line treatment with mitomycin-based regimens. Current evidence indicate that the use of 5-FU, oxaliplatin and irinotecan at any sequence within patient's care pathway has survival advantages (Grothey *et al*, 2004). The predominant treatment pathways showed that NHS clinical practice reflects the best available evidence on clinical and cost-effectiveness, despite a paucity of guidelines outlining best practice and individual clinicians having to rely on their own clinical experience and personal interpretation of current evidence to guide practice. It was interesting to note that out of 22 trials identified that investigated the use of irinotecan with 5-FU, only 1 used IrMdG, the UK-relevant regimen. This implies that clinical practice within UK CRC specialties is atypical compared to other countries. It also points to the scarcity of data guiding UK practice.

Data from the sequential trials (Tournigand *et al*, 2004; Seymour *et al*, 2007) revealed that combination irinotecan regimens have higher frequencies of toxicity in the first-line setting compared to second-line setting, potentially indicating that only fitter trial patients received second-line chemotherapy. Comparing the frequencies of adverse events from the RCTs (Table 1) with NHS experts' estimates (Table 3) revealed that irinotecan-related febrile neutropaenia occurs more frequently within NHS settings. The frequency of irinotecan-related diarrhoea in the second-line setting was also higher in NHS practice than in the trials. Differences between the frequencies of adverse events in NHS practice and the trials may be due to variations in patient demographics, local clinical practice or chemotherapy treatment patterns. A National Institute for Health Clinical Excellence (NICE) health technology assessment (HTA) (National Institute for Health Clinical Excellence, 2005) highlighted concern in extrapolating trial data (Tournigand *et al*, 2004; Seymour *et al*, 2007) to NHS patients because the trial populations were relatively young and fit compared to UK CRC population. This suggests that the safety profile of irinotecan regimens may be exaggerated as younger and fitter patients may be less likely to experience clinically significant adverse events, such as febrile and septic neutropaenia. Furthermore, prescriptive trial protocols often determine clinical management, such as patient monitoring, follow-up intervals, prescribing patterns and management of ADEs, and do not reflect NHS practice.

Differences around experts' estimates may reflect their uncertainty of the true frequencies of ADEs occurring in NHS practice. Furthermore, differences in the estimates may be a reflection of variations between institutions and regions, such as different funding resources, local treatment protocols and individual clinician's prescribing preferences. Frequencies of ADEs reduced when estimates were weighted with number of advanced CRC patients seen in 2007, potentially indicating that either clinicians treating many CRC patients are more confident in irinotecan's safety profile or increased experience with chemotherapy agents leads to reduced adverse events. The clinicians' low estimate for neutropaenia-related deaths indicated that this is a rare event. This may suggest that irinotecan-based regimens are relatively safe and neutropaenic episodes are well managed, resulting in low mortality rate. However, it may also be influenced by the nature of NHS patient referral pathways, where patients may seek treatment for chemotherapy-related complications in a different NHS institution than where chemotherapy was administered. The oncology team administering chemotherapy may not be aware of patient's chemotherapy-related morbidity or mortality. A recent study (National Confidential Enquiry into Patient Outcome and Death, 2008) found that 15% of cancer patients hospitalised during their last 30 days of life were not admitted into the same institution where their systemic chemotherapy was administered.

More than half of the consultants (52%) reported using G-CSF in their NHS practice. The main indications reported for G-CSF use were septic neutropaenia and secondary prophylaxis. ASCO recommends G-CSF as secondary prophylaxis (Smith *et al*, 2006) *in situations* where chemotherapy dose intensity must be preserved to optimise cytotoxic drugs exposure, implying that patients with previous neutropaenia are potentially more resource intensive. Few clinicians used G-CSF to treat febrile neutropaenia, in line with EORTC (Aapro *et al*, 2006) and ASCO (Smith *et al*, 2006) recommendations that G-CSF should only be considered when patients present septic symptoms. In October 2009, the Department of Health requested NICE to prepare a clinical guideline on the prevention and management of neutropaenic sepsis in cancer patients, underlining the need for clear UK-relevant recommendations (National Institute of Health and Clinical Excellence, 2009). This report is yet to be published.

Use of UGT1A1 pharmacogenetic testing in CRC specialties is currently low, with an increase in demand predicted as many clinicians indicated they would like to use the test. Pharmacogenetic tests will only optimise patients' outcome if prescribing is guided by test results. This study did not explore how clinicians would change their clinical practice based on information from the UGT1A1 test. Without a regulatory body providing clear recommendations on how pharmacogenetic test information can be used to alter clinical pathways, clinicians' demand for a pharmacogenetic test may not translate into changes in their practice or improvement in patient benefits. It is difficult to predict how CRC oncologists will use UGT1A1 test results in practice and its subsequent impact on patients' pathways and clinical outcome. Wide variations in CRC clinical practice are potentially associated with variations in the application of pharmacogenetic testing within the specialty, representing additional challenges in assessing the added value of a test whose main objective is to inform and change current prescribing practice. This offers a further challenge in assessing the added value of such companion diagnostic tests early in their development.

### Limitations

This study used expert opinion in the absence of other data sources. A detailed retrospective chart review or a large prospective observational study would be ideal to capture treatment pathways, clinical management and frequency of adverse events within NHS CRC specialties. However, there are several challenges to conducting these types of studies in the United Kingdom. First, the exact number of CRC consultants in the United Kingdom is not known and there is no means of identifying these consultants. Second, patients' medical records in the United Kingdom are not fully computerised and data extraction needs to be carried out manually for each individual patient at the respective health-care institutions across the country. This means that such a study would require considerable time, manpower and funding, and the results would not be timely. Due to barriers to conducting such a costly study, this pragmatic study was designed.

This study used a postal survey as a systematic method to elicit expert opinion in a clinical specialty with wide variations in prescribing practice. Further research is needed to directly compare this method to other methods that use smaller sample sizes and are potentially more resource intensive, such as Delphi methods (Antonini *et al*, 2008) and methods using specialised software (Garthwaite *et al*, 2008). However, consensus-generating Delphi may introduce additional challenges, as it may not adequately incorporate uncertainty.

Care must be taken when generalising the results of this survey to other jurisdictions. The sample size of the expert panel is 44 consultants and potentially represents a good proportion of eligible consultants in England. The actual number of eligible consultants is not known as there is no single database of NHS CRC oncologists. Assuming that all of the 178 NHS Hospital Trusts have one CRC oncology consultant each, the potential total sample is 178 consultants. A NICE HTA previously estimated that there are 12 665 advanced CRC patients in England and Wales receiving chemotherapy annually (National Institute for Health and Clinical Excellence, 2005). Between the 44 consultants, they treated an estimated 5940 patients in 2007. Previous elicitation studies that generated parameter estimates for economic models used smaller numbers of experts (*n*=3 (Garthwaite *et al*, 2008) and *n*=6 (Leal *et al*, 2007)) and had identified the experts beforehand. Due to the study's main aim to describe CRC clinical practice, with a focus on irinotecan prescribing practice, the strict inclusion criteria may have resulted in a small population of interest. Data obtained may only reflect the views of this specific group of consultants but there were no systematic differences found between respondents and the non-respondents contacted in the telephone follow-up.

### Implications

Wide variations identified in the chemotherapy pathways of CRC patients are not uncommon within oncology specialties, and may occur in other clinical specialties with a fast changing evidence base. Similar variations exist in the treatment pathway of chemotherapy-induced neutropaenia. These variations may have clinical implications on patients' morbidity and mortality. There is a need to strike the right balance between allowing clinicians to decide based on their judgement and having prescriptive clinical guidelines.

The findings from this study will inform an economic evaluation of UGT1A1 pharmacogenetic test, which will ensure that the economic analysis is relevant to UK clinical practice and useful to NHS decision-makers. Data on the main chemotherapy treatment pathways for advanced CRC patients will be used to inform the model structure and the selection of the UK-relevant irinotecan-based regimen. Data on the management of patients on irinotecan-based chemotherapy and the clinical management of irinotecan-related neutropaenia will be used to ensure that the estimated NHS resource use downstream of the intervention assessed is relevant to current practice. The ranges and distribution around the clinicians' estimates on the frequencies of adverse events will be used in a probabilistic sensitivity analysis to explore the impact of the uncertainty around the point estimates.

This study showed the use of critical evidence synthesis and elicitation of expert opinion to inform the design of an economic evaluation. The results provide externally valid information and descriptive empirical evidence upon which to structure the evaluation. Similar methods can also be used to inform technology assessments for reimbursement decisions, for example in HTAs and appraisals of chemotherapy drugs. Current practice in oncology is often not fully understood because of the rapidly evolving evidence base and variations in treatment pathways, and this affects the evaluations in defining the research question, scope of the evaluation and subsequent analysis, as well as relevance of results to NHS practice and to decision-makers. For example, NICE HTA for bevacizumab used irinotecan with bolus 5-FU (IFL regimen) as the comparator for first-line treatment of advanced CRC (National Institute of Health and Clinical Excellence, 2006). Further understanding of NHS clinical practice through this study has revealed that irinotecan regimens are not relevant comparators in first-line setting where oxaliplatin-based regimens are the main chemotherapy of choice; and the IFL regimen is not used in NHS CRC practice. Selecting a relevant comparator is a key aspect of ensuring an economic evaluation provides useful information for decision-making. Inappropriate decisions regarding the choice of comparator could become a source of uncertainty (Bojke *et al*, 2009) and can bias the estimated added value of new treatments.

## Conclusion

This study describes variations in NHS prescribing practice and clinical management of advanced CRC patients receiving palliative chemotherapy. Irinotecan-based regimens were primarily used as second-line chemotherapy. The two main regimens are irinotecan with infusional 5-FU (IrMdG) and irinotecan monotherapy. Experts estimated that while uncomplicated neutropaenia is common, clinically significant neutropaenic events are less frequent. This method of eliciting expert opinion could potentially capture heterogeneity in practice and show the complexity of modelling a standard patient pathway for clinical specialties that have wide variations in practice with rapidly changing prescribing and clinical practice.

## Figures and Tables

**Table 1:** Frequency of irinotecan-related neutropaenia and diarrhoea occurring in clinical trials

**Study (Country)**	**Seymour *et al*, 2007** **(United Kingdom)**		**Tournigand *et al*, 2004** **(France)**		**Van Cutsem *et al*, 2009** **(Multinational trial)**
Regimen	IrMdG: irinotecan 180 mg m^−2^ (30 min), levofolinate 175 mg (2 h), 5-FU 400 mg m^−2^ (bolus) and 5-FU 2400 mg m^−2^ (46 h)		FOLFIRI: L-LV 200 or DL-LV 400 mg m^−2^ (2 h), irinotecan 180 mg m^−2^ (90 min), 5-FU 400 mg m^−2^ (bolus) and 5-FU 2400–3000 mg m^−2^ (46 h)		FOLFIRI: L-LV 200 or DL-LV 400 mg m^−2^ (2 h), irinotecan 180 mg m^−2^ (30–90 min), 5-FU 400 mg m^−2^ (bolus) and 5-FU 2400 mg m^−2^ (46 h)
Previous chemo	None	5-FU	None	FOLFOX6	None
					
Adverse drug events	Mean (%)	Mean (%)	Mean (%)	Mean (%)	Mean (%)
Neutropaenia (all grades)	Not reported	Not reported	76 (all grades)	60 (all grades)	Not reported
Neutropaenia (G3 & G4)	19 (G3 & G4)	18 (G3 & G4)	15 (G3) and 9 (G4)	21 (G3) and 0 (G4)	25 (G3 & G4)
Febrile neutropaenia	Not reported	Not reported	0 (G1 & G2) and 7 (G3 & G4)	0 (G1 & G2) and 1 (G3 & G4)	2 (G3 & G4)
Severe diarrhoea	12 (G3 & G4)	8 (G3 & G4)	9 (G3) and 5 (G4)	7 (G3) and 1 (G4)	11 (G3 & G4)

Abbreviations: 5-FU=5-fluorouracil; IrMdG=irinotecan modified de Gramont; LV=leucovorin.

**Table 2:** Frequently used first-, second- and third-line chemotherapy regimens for advanced colorectal cancer patients

	**Mean percentage of use**	
**Chemotherapy regimen** **First-line chemotherapy**	**Unweighted mean^a^ (%)**	**Weighted mean^b^ (%)**
Capecitabine+oxaliplatin	37.5	41.4
Oxaliplatin+MdG	22.7	26.5
Capecitabine or UFT	24.3	17.6
FOLFOX	4.9	4.9
Irinotecan+MdG	3.8	4.5
		
**Second-line chemotherapy**	**Unweighted mean^c^ (%)**	**Weighted mean^d^ (%)**
Irinotecan+MdG	23.2	30.5
Single agent irinotecan	21.4	25.9
Capecitabine+oxaliplatin	12.1	11.0
FOLFIRI	11.9	7.3
Oxaliplatin+MdG	9.0	6.7
		
**Third-line chemotherapy**	**Unweighted mean^e^ (%)**	**Weighted mean^f^ (%)**
Mitomycin+5-FU or M+Cap	49.0	44.6
Capecitabine or UFT	11.9	9.2
Capecitabine+irinotecan	7.1	7.0
5-FU regimen	6.7	13.9
Single agent irinotecan	6.5	15.0

Abbreviations: 5-FU=5-fluorouracil; MdG=modified de Gramont.

Number of respondents, *n*.

a *n*=42.

b *n*=41.

c *n*=40.

d *n*=39.

e *n*=24.

f *n*=23.

**Table 3:** Frequency of irinotecan-related neutropaenia and diarrhoea estimated by NHS consultant oncologists

**Study**	**Experts' estimates (from survey)**			
**Regimen**	**Second-line IrMdG**			
**Adverse drug events**	**Unweighted**			**Weighted**
	**Mean (%) (s.d.)**	**Median (mode)**	**95% CI (ranges)**	**Mean (%)**
Uncomplicated Neutropaenia	32.9 (17.5)	30 (20)	27.4–38.3 (10.0–80.0)	27.2
Febrile neutropaenia	8.4 (5.3)	5 (5)	6.7–10.0 (2.5–25.0)	7.8
Septic neutropaenia	4.7 (4.3)	4 (5)	3.4–6.0 (0.5–20.0)	3.8
Severe diarrhoea	13.1 (7.7)	10 (10)	10.8–15.5 (1.0–30.0)	12.7

Abbreviation: IrMdG=irinotecan modified de Gramont.
